# Effect of nanoparticles on the *ex-vitro* performance of cryopreservation-derived plant material

**DOI:** 10.1371/journal.pone.0310424

**Published:** 2024-09-12

**Authors:** Dariusz Kulus, Alicja Tymoszuk, Alicja Kulpińska, Iva Viehmannova, Jacek Wojnarowicz, Urszula Szałaj

**Affiliations:** 1 Laboratory of Horticulture, Faculty of Agriculture and Biotechnology, Bydgoszcz University of Science and Technology, Bydgoszcz, Poland; 2 Department of Crop Sciences and Agroforestry, Faculty of Tropical AgriSciences, Czech University of Life Sciences Prague, Prague, Czech Republic; 3 Laboratory of Nanostructures, Institute of High Pressure Physics, Polish Academy of Sciences, Warsaw, Poland; Saveetha Institute of Medical and Technical Sciences: Saveetha University, INDIA

## Abstract

The integration of nanoparticles into plant cryopreservation protocols holds great promise for improving the survival rates and recovery potential of explants. This study aimed to verify the effect of nanoparticles on the *ex-vitro* performance of cryopreservation-derived plants. *Lamprocapnos spectabilis* (L.) Fukuhara (bleeding heart) ’Gold Heart’ and ’Valentine’ cultivars were used as the plant material. The encapsulation-vitrification cryopreservation protocol of shoot tips included the preculture, encapsulation, dehydration, storage in liquid nitrogen, rewarming, and recovery steps. Gold (AuNPs), silver (AgNPs), or zinc oxide (ZnONPs) nanoparticles were added at varying concentrations, either into the preculture medium or the protective bead matrix during encapsulation. After the *in vitro* recovery, the plants were transferred to the glasshouse and subjected to detailed biometrical, biochemical and cytogenetic analyses. Nanoparticles had no evident effect on the acclimatization efficiency (80–100% survival) and leaf number in *L*. *spectabilis* ‘Gold Heart’. Nonetheless, shoots developed from alginate beads supplemented with 5 ppm AuNPs were twice as long as the control, while the leaves of plants grown on the preculture medium with ZnONPs contained significantly more chlorophyll and had higher Leaf Soil-Plant Analysis Development (SPAD) values. Moreover, several NPs treatments stimulated the development of leaves, including their surface area, length, and perimeter. Higher ZnONPs levels enhanced also the replication process, resulting in higher nuclear DNA content. As for *L*. *spectabilis* ‘Valentine’, alginate augmentation with 5 ppm AgNPs or 5 ppm ZnONPs stimulated the elongation of shoots. There was also a tendency suggesting a positive influence of 5 ppm AgNPs in the alginate bead matrix on foliar growth. The effect of nanoparticles on the content of flavonoids, anthocyanins, and stress markers in the plants varied depending on the treatment and cultivar, but also on the organ studied (leaf or stem). Overall, *L*. *spectabilis* ‘Gold Heart’ was more stress-tolerant and genetically stable than ‘Valentine’ judging by the activity of Photosystem II (PSII) and flow cytometric analyses, respectively. The complex effects of nanoparticles on survival, biometric parameters, physiological responses, and cytogenetic events underscore the intricate interplay between nanoparticles and plant systems. Nonetheless, our research confirmed the positive effect of nanoparticles on the *ex-vitro* growth and development of *L*. *spectabilis* plants after cryostorage.

## Introduction

The application of nanoparticles (NPs) in plant biotechnology has emerged as a cutting-edge and innovative field with significant implications for agricultural sustainability and crop improvement. Nanoparticles, characterized by their small size (below 100 nm) and unique physicochemical properties, can be applied to enhance various aspects of plant biology [1]. From nanoparticle-mediated delivery of nutrients and agrochemicals to the development of nanomaterial-based sensors for real-time monitoring of plant health and environmental conditions, this rapidly evolving discipline presents novel solutions to long-standing challenges in agriculture [2].

The utilization of NPs in plant tissue culture offers a range of innovative applications and benefits. Nanoparticles have been integrated into plant tissue culture protocols to enhance and optimize various aspects of the process [3]. These tiny structures have proven effective in improving the growth and development of plant tissues, from the micropropagation of elite cultivars [4] to the genetic transformation of recalcitrant species [5].

In micropropagation, the controlled release of growth regulators and nutrients via nanoparticle carriers has allowed for more precise and sustained delivery to cultured plant tissues, resulting in enhanced shoot multiplication rates, root development, and overall growth performance [3]. Gold nanoparticles (AuNPs) promoted the *in vitro* proliferation of *Nardostachys jatamansi* DC [6]. Furthermore, NPs have been employed to alleviate issues associated with hyperhydricity syndrome, a common physiological disorder in tissue culture, by regulating the water status and hormonal balance in plant explants [7]. This aids in the production of healthier and more acclimatization-ready plantlets. Zinc oxide nanoparticles (ZnONPs) were used to modify the architecture of *in vitro*-propagated plants of *Chrysanthemum × morifolium* (Ramat.) Hemsl. [8] and their biochemical profile [9]. On the other hand, silver nanoparticles (AgNPs) were utilized in mutation breeding due to their genotoxic effects if applied at higher concentrations [10]. The use of nanoparticles in plant tissue culture has not only advanced the efficiency of micropropagation and breeding techniques but also raised intriguing questions regarding their potential use in long-term storage and cryopreservation.

Cryopreservation, the process of preserving biological materials at ultra-low temperatures of liquid nitrogen (LN; -196°C), is a critical technique in various fields, including biotechnology and agriculture [11]. Climate change, habitat loss and population growth are a significant threat to plant species and agricultural diversity. Cryopreservation offers a reliable method for preserving clonal germplasm, endangered species, and crop wild relatives. By storing plant tissues at ultra-low temperatures, metabolic activities are arrested, allowing for the preservation of genetic material for extended periods [12]. As such, advancements in cryopreservation techniques are essential for the resilience of agricultural systems worldwide. Conventional cryopreservation methods mainly rely on the use of cryoprotectants (CPAs) that are able to penetrate the cell membrane, such as dimethyl sulfoxide (DMSO) and ethylene glycol. However, these CPAs (DMSO especially) have been shown to be toxic, causing adverse reactions in cells [12, 13]. Therefore, it is of great interest to improve the existing cryo-protocols by substituting aggressive CPAs with more effective materials. Recent discoveries in nanotechnology have triggered interest in the application of nanoparticles to enhance the efficiency and success of cryopreservation [14].

Nanoparticles have been harnessed as cryoprotectants and ice modulators, mitigating the formation of ice crystals and minimizing cellular damage during the freezing and thawing process in animal cells [15]. Their small size allows them to penetrate cell membranes and interact with intracellular components, safeguarding the structural and functional integrity of biological materials [16]. Furthermore, nanoparticles can serve as carriers for various bioactive molecules, such as cryoprotectants, offering their controlled and targeted delivery to the cells prior to cryopreservation. This approach not only enhanced semen survival but also reduced the toxicity associated with conventional cryoprotectants [17].

While the potential benefits of nanoparticle-assisted cryopreservation are applied with animal cells for cryopreservation of gametes and embryos [18, 19], their use with plant tissues is highly reduced. It was reported with *L*. *spectabilis* (L.) Fukuhara ‘Valentine’ that the efficiency of shoot tip cryopreservation via encapsulation-vitrification can be elevated by even 20% after adding 13-nm AuNPs at 10 ppm concentration into the alginate bead matrix [20]. On the other hand, the ‘Gold Heart’ cultivar benefited from alginate supplementation with 5 ppm AgNPs and 5–15 ppm ZnONPs, leading to an over 28% increase in the survival rate of shoot tips [21]. Therefore, deeper research in this regard is necessary. It is particularly important to address concerns about the long-term effects of nanoparticles within cryopreserved biological systems. Numerous studies reported the effect of cryopreservation on the field performance of LN-derived plants [22, 23]. In most of them, no significant changes in growth and morphology were observed as it is assumed that cryopreservation generally does not affect the properties of plants [22]. Some researchers, however, showed interesting alterations in the plant material. For example, reduced seed germination was observed in the cryopreserved seeds of *Zea mays* and *Glycine max* [24, 25]. Seed cryopreservation decreased Cd, Cu and Na uptake and increased the absorption of Al and B elements in the cryo-derived seedling of *Phaseolus vulgaris* L. [26]. While cryopreservation-derived plants exhibited some reduction in root formation and vegetative growth, the quantity and quality of flowers remained comparable between cryo- and *in vitro*-derived plants of the perennial ornamental species *Argyranthemum maderense* (D. Don) Humphries [27]. Likewise, despite cryopreservation by encapsulation-dehydration did not disturb the chimeric structure of *C*. *morifolium* albeit it affected its growth (elongation of shoots, size of leaves and flowering time) in a cultivar-dependent matter [28].

There are also studies reporting the significant impact, both positive and negative, of nanoparticles on the field growth of plants, depending on (among others) the species, type, size, shape and concentration of NPs [29]. For instance, spraying 10 ppm ZnONPs on leaves promoted the growth and biomass accumulation of *Coffea arabica* L. plants [30]. A low concentration of iron nanoparticles (FeNPs) could stimulate the growth of *Capsicum annuum* L. seedlings [31], while AuNPs positively influenced all growth parameters of *Arabidopsis* seedlings [32]. Single-walled carbon nanotubes (SWCNT), significantly enhanced photosynthetic yield in chloroplasts, achieving a three-fold increase due to their ability to facilitate rapid electron transport and boost the activity of plant signaling molecules like nitric oxide [33]. Additionally, nanoparticles, such as silicon oxide (SiO_2_NPs), cuprum (CuNPs), iron (FeNPs), molybdenum (MnNPs), and potassium (KNPs), enable plants to better adapt to stressful conditions and increase their yields by mitigating the toxic effects of stressors and influencing various morphological, anatomical, physiological, biochemical, and molecular attributes of plants [34]. In contrast, high concentrations (50, 100 and 200 ppm) of ZnONPs, and AgNPs led to leaf size reduction, chlorosis and lateral roots inhibition in *Arabidopsis thaliana* (L.) Heynh. [35] and *Cucumis sativus* L. seedlings [36]. To date, there is no information on the *ex vitro* performance of plant material cryopreserved with the use of nanomaterials. There is also little research evaluating the simultaneous effect of various types of nanoparticles on different plant cultivars. As such, continued research is essential to understand the full scope of nanoparticle interactions within plant systems and to ensure the safe and responsible integration of these nanomaterials in agricultural practices.

The aim of this study was to investigate the impact of AuNPs, AgNPs, and ZnONPs, applied at various concentrations at two steps of the encapsulation-vitrification protocol (preculture and encapsulation) on the *ex-vitro* performance of LN-derived *Lamprocapnos spectabilis* ’Gold Heart’ and ’Valentine’ cultivars. This popular perennial of the Fumarioideae subfamily (Papaveraceae) is prized both as an ornamental (indoor/outdoor, pot and cut plant) and medicinal-cosmetic species [37]. The study focused on assessing the acclimatization survival rates, detailed biometric parameters, physiological responses, and cytogenetic events of *L*. *spectabilis* plants after cryostorage and transplantation to the glasshouse. The hypothesis assumed that the addition of specific nanoparticles at optimized concentrations during cryopreservation can enhance the cryopreservation efficiency in terms of plant growth and development *ex vitro*.

## Materials and methods

### Cryopreservation procedure

*In vitro*-derived microshoots of *Lamprocapnos spectabilis* (L.) Fukuhara ‘Gold Heart’ and ‘Valentine’ were used as the source of explants. The cryopreservation procedure consisted of several sequential steps, including preculture, encapsulation, dehydration/vitrification, low-temperature (LN) storage, rewarming, and recovery, according to the previously developed protocol [38].

### Experiment I—Effect of NPs in the preculture medium

Single-node explants were cultured *in vitro* for one week on a solid MS medium [39] containing 9% (*w/v*) sucrose, 4.65 μM (1.0 mg L^-1^) kinetin (KIN), and 10 μM (2.62 mg L^-1^) abscisic acid (ABA). Ten explants were placed in each culture vessel (glass jar) filled with 30 mL of medium. The medium was sterilized at 121°C for 20 min.

Silver, gold (6 nm in diameter), or zinc oxide (13 nm) nanoparticles, at concentrations of 5 and 15 ppm, were distributed equally, through a sterile filter (0.22 μm pore size), on the culture medium surface immediately after explant inoculation (2 mL per jar). The control group consisted of non-treated explants. The cultures were kept in a growth room at 24°C ± 1°C, under 16-h photoperiod conditions and photosynthetic photon flux density of approximately 30.0 μmol m^-2^ s^-1^ provided by standard cool daylight TLD 54/36W fluorescent lamps (Koninklijke Philips Electronics N.V., Eindhoven, The Netherlands).

After one week, shoot tips (1.0–2.0 mm long) were excised and encapsulated in a 3% (*w/v*) sodium alginate solution based on MS medium salts without CaCl_2_, supplemented with 9% sucrose. Beads were hardened in 0.1 M CaCl_2_ for 30 min, rinsed, osmoprotected with a loading solution (2.0 M glycerol and 0.4 M sucrose), and dehydrated with Plant Vitrification Solution 3 (PVS3; 50% glycerol and 50% sucrose, w/v) for 150 min. Ten beads covered with PVS3 were placed in a 2.0 mL sterile cryovial and directly immersed in LN.

After a day in LN storage, cryovials were rapidly rewarmed in a water bath, and explants were rinsed in liquid MS medium with 1.2 M sucrose (for 30 min) and inoculated on the MS recovery medium with 3% sucrose and 2.22 μM (0.5 mg L^-1^) 6-benzyladenine (BA). The cultures were kept in the same growth room for 60 days (in total darkness for the first two days).

### Experiment II—Effect of NPs in the alginate matrix

The same cryopreservation steps and parameters were used as in Experiment I, with the distinction that no NPs were added to the preculture medium. Instead, silver (AgNPs), gold (AuNPs), and zinc oxide (ZnONPs) nanoparticles at 5 ppm and 15 ppm were incorporated into the sodium alginate solution during encapsulation. A control group with no nanoparticles was also included in this experiment.

### Acclimatization and *ex-vitro* growth

The rooted microshoots were transferred to the greenhouse (53°07’12.0"N 18°00’29.4"E) in June for acclimatization and further growth in natural light conditions (20 per experimental treatment). Plants of *L*. *spectabilis* were planted in plastic trays filled with a mixture of peat and perlite (2:1) provided by Hartmann (Poznań, Poland), sprayed with water for two weeks, and covered with perforated transparent foil. The acclimatization effectiveness (survival rate) was assessed after 14 days. One month after transferring to *ex vitro* conditions, the plants were transplanted to plastic pots filled with the same substrate and planted in a permanent place on benches for another 30 days (S1 Fig).

### Biometrical analysis of plants

Two months after transferring to the glasshouse, the phenotype analysis of plants was performed visually and with the use of the Royal Horticultural Society Colour Chart (RHSCC) key [40] in natural light conditions (10 plants per treatment). The length of the shoots was measured and the number of leaves was counted. By applying the WinFolia 2016b and XLFolia 2016a software (Regent Instruments, Quebec, Canada) and the EPSON Perfection V800 Photo scanner, the leaf architecture; including the total area, perimeter, length, maximum and average width were measured. Based on that data, the aspect ratio (the ratio of horizontal width to vertical length) and the form coefficient (a value that grades the leaf shape as between the shortest and longest perimeter for a given area) were counted. To evaluate leaf architecture, three leaves were randomly selected from each plant.

### Physiological array: Stress effects and determination of pigments in leaves and stems

The relative content of flavonoids, anthocyanins, chlorophyll content index (CCI) and Leaf Soil-Plant Analysis Development (SPAD) value in the leaves and stems was measured in triplicates in each plant using an MPM-100 multi-pigment meter (Opti-Sciences Inc, Hudson, NH, USA).

The level of stress was measured based on the maximum efficiency of photosystem II (PSII) in leaves of at least five plants. The fluorescence kinetics of chlorophyll was measured using a portable plant stress meter OS30p+ (Opti-Sciences Inc) and then initiative fluorescence (F0), variable fluorescence (Fv), maximum fluorescence (Fm), and their ratios (Fv/Fm and Fv/F0) were determined and expressed in relative units.

### Estimation of nuclear DNA content

The 2C DNA content was estimated using flow cytometry (FCM) in 260 plants (10 plants per treatment, each plant was considered a single replication). The analysis was performed on sampled young leaves. All samples for the FCM analysis were prepared according to two-step procedure by [41] using Otto I buffer (0.1 M citric acid, 0.5% Tween 20) and Otto II buffer (0.4 M Na_2_HPO_4_ 12H_2_O) supplemented with propidium iodide (PI) as fluorescent dye, RNAse IIA (both at final concentrations of 50 μg mL^-1^) and β-mercaptoethanol (2 μg mL^-1^). As an internal standard, *Solanum lycopersicum* ´Stupicke´ (2C = 1.96 pg) [42] was used. For each sample, at least 5,000 nuclei were measured using a CyFlow Space C32 flow cytometer equipped with a green solid-state laser (532 nm, 100 mW) as an excitation source (Sysmex Partec GmbH, Görlitz, Germany). The histograms were evaluated using the FlowMax software (Sysmex Partec GmbH, Görlitz, Germany). The nuclear DNA content was calculated as the ratio of 2C peaks of the sample and the internal standard, multiplied by the genome size of the internal standard.

### Statistical analysis

The experiments were arranged in a completely randomized design for two cultivars separately. Each of the two experiments, involving the application of nanoparticles during the preculture (*prec*) or encapsulation (*enc*) step, comprised seven treatments: control, 5 ppm AgNPs, 15 ppm AgNPs, 5 ppm AuNPs, 15 ppm AuNPs, 5 ppm ZnONPs, and 15 ppm ZnONPs.

The obtained results underwent statistical analysis through one-way ANOVA, and mean comparisons were conducted using Duncan’s Test (P ≤ 0.05) with Statistica 12.0 (StatSoft, Poland) and ANALWAR-5.2-FR tools. The arcsine transformation was done on data expressed as a percentage.

## Results

### Effect of nanoparticles on the *ex vitro* performance of LN-derived plants

There was no impact of nanoparticles neither on the survival of *L*. *spectabilis* ‘Gold Heart’ during acclimatization (80–100%) nor on the number of leaves produced (6.8–11.4). Nonetheless, shoots developed from alginate beads supplemented with 5 ppm AuNPs were two-fold longer than the control.

As for *L*. *spectabilis* ‘Valentine’, the addition of 15 ppm ZnONPs into the preculture medium affected adversely the acclimatization efficiency (60% survival) compared to most other treatments and the control (90–100%). Alginate supplementation with 5 ppm AgNPs or 5 ppm ZnONPs stimulated the elongation of shoots that were twice as long as the control. On the other hand, explants precultured on the medium with 15 ppm ZnONPs produced the lowest number of leaves (4.6) (Table 1).

**Table 1:** Effect of silver (AgNPs), gold (AuNPs), and zinc oxide (ZnONPs) nanoparticles applied during the preculture (prec) or encapsulation (enc) step of the encapsulation-vitrification cryopreservation protocol on the survival of plants after 14 days of acclimatization, as well the length of shoots, number of leaves, and the dominating leaf color (RHSCC) after 60 days of *ex vitro* growth in *Lamprocapnos spectabilis* ‘Gold Heart’ and ‘Valentine’.

	Survial (%)		Shoot length (cm)		No. of leaves		RHSCC
**Treatment**	**Gold Heart**						
**control**	100 ± 0	a	37.1 ± 5.22	b-d	9.0 ± 1.51	a	150a
**5 ppm AgNPs prec**	100 ± 0	a	51.1 ± 6.77	ab	9.7 ± 1.12	a	150a
**15 ppm AgNPs prec**	100 ± 0	a	36.7 ± 4.44	b-d	8.1 ± 0.48	a	150a
**5 ppm AuNPs prec**	100 ± 0	a	25.2 ± 3.29	d	7.3 ± 1.07	a	150a
**15 ppm AuNPs prec**	100 ± 0	a	31.2 ± 4.85	cd	8.6 ± 1.08	a	150a
**5 ppm ZnONPs prec**	90 ± 10.0	a	34.0 ± 4.49	b-d	7.7 ± 0.72	a	150a
**15 ppm ZnONPs prec**	80 ± 13.3	a	25.1 ± 4.07	d	7.0 ± 1.08	a	150a
**5 ppm AgNPs enc**	100 ± 0	a	46.0 ± 6.81	bc	11.4 ± 1.66	a	150a
**15 ppm AgNPs enc**	100 ± 0	a	39.5 ± 6.02	b-d	9.4 ± 0.95	a	150a
**5 ppm AuNPs enc**	100 ± 0	a	66.0 ± 10.47	a	9.6 ± 1.35	a	150a
**15 ppm AuNPs enc**	100 ± 0	a	27.1 ± 2.58	c	6.8 ± 0.54	a	150a
**5 ppm ZnONPs enc**	100 ± 0	a	30.7 ± 4.69	cd	7.9 ± 0.84	a	150a
**15 ppm ZnONPs enc**	85.7 ± 9.7	a	29.1 ± 4.85	cd	8.0 ± 1.50	a	150a
	**Valentine**						
**control**	100 ± 0	a	31.9 ± 5.07	b-e	11.9 ± 1.98	a-c	143a
**5 ppm AgNPs prec**	90 ± 10.0	a	23.7 ± 1.96	c-e	8.4 ± 0.41	c-e	143a
**15 ppm AgNPs prec**	90 ± 10.0	a	21.9 ± 1.82	de	5.9 ± 0.42	de	143a
**5 ppm AuNPs prec**	100 ± 0	a	31.0 ± 4.67	b-e	11.2 ± 1.37	a-d	143a
**15 ppm AuNPs prec**	90.9 ± 9.1	a	17.7 ± 2.11	e	9.0 ± 1.26	b-e	143a
**5 ppm ZnONPs prec**	90 ± 10.0	a	30.4 ± 8.51	b-e	7.4 ± 1.30	c-e	143a
**15 ppm ZnONPs prec**	60 ± 16.3	b	19.7 ± 2.72	e	4.6 ± 1.21	e	143a
**5 ppm AgNPs enc**	100 ± 0	a	65.4 ± 8.57	a	14.4 ± 1.78	ab	143a
**15 ppm AgNPs enc**	90 ± 10.0	a	47.3 ± 7.85	ab	9.3 ± 1.36	a-e	143a
**5 ppm AuNPs enc**	85.7 ± 9.7	ab	44.5 ± 8.26	a-d	12.4 ± 2.36	a-c	143a
**15 ppm AuNPs enc**	84.6 ± 10.4	ab	30.2 ± 8.91	b-e	8.4 ± 0.59	c-e	143a
**5 ppm ZnONPs enc**	100 ± 0	a	64.7 ± 10.57	a	14.5 ± 2.17	a	143a
**15 ppm ZnONPs enc**	92.3 ± 10.0	a	45.8 ± 8.71	a-c	11.0 ± 2.14	a-d	143a

* Each number represents the mean value ± standard error. Significant differences in values are determined by Duncan’s *post hoc* test (*P*<0.05). Values with at least one same letter in column are not statistically different.

The leaves of ‘Gold Heart’ plants were in general yellow-green in color (150a RHSCC code), while ‘Valentine’ plants were predominantly dark green (143a) (Table 1).

The lowest values of leaf biometric parameters (i.e. the surface area, perimeter, length, maximal and average width) in cv. ‘Gold Heart’ were found in the untreated control (Table 2). On the other hand, several NPs treatments stimulated the development of leaves, with 5 ppm AgNPs/ZnONPs in the preculture medium or 5 ppm AuNPs in the alginate bead being the most effective. No significant changes in the leaf shape, determined by the aspect ratio and form coefficient, were found (Table 2).

**Table 2:** Effect of silver (AgNPs), gold (AuNPs), and zinc oxide (ZnONPs) nanoparticles applied during the preculture (prec) or encapsulation (enc) step of the encapsulation-vitrification cryopreservation protocol on the leaf blade parameters in shoots after 60 days of *ex vitro* growth in *Lamprocapnos spectabilis* ‘Gold Heart’ and ‘Valentine’.

	**Area (cm** ^**2**^ **)**		**Perimeter (cm)**		**Length (cm)**		**Max. width (cm)**	
**Treatment**	**Gold Heart**							
**control**	13.8 ± 2.11	d	40.0 ± 3.96	e	7.2 ± 0.77	c	5.0 ± 0.44	d
**5 ppm AgNPs prec**	26.3 ± 3.21	a	58.8 ± 3.86	a	9.1 ± 0.68	a-c	8.0 ± 0.56	ab
**15 ppm AgNPs prec**	23.0 ± 2.56	ab	54.6 ± 3.80	a-d	9.6 ± 0.74	ab	6.9 ± 0.52	a-c
**5 ppm AuNPs prec**	22.1 ± 1.89	a-c	54.6 ± 2.53	a-d	9.3 ± 0.51	a-c	7.2 ± 0.38	a-c
**15 ppm AuNPs prec**	20.5 ± 2.24	a-d	51.6 ± 3.25	a-d	8.9 ± 0.59	a-c	6.5 ± 0.46	b-d
**5 ppm ZnONPs prec**	25.7 ± 2.51	a	57.9 ± 3.97	ab	10.1 ± 0.70	a	6.9 ± 0.43	a-c
**15 ppm ZnONPs prec**	14.6 ± 1.57	de	44.0 ± 2.99	de	8.0 ± 0.52	a-c	5.7 ± 0.38	de
**5 ppm AgNPs enc**	20.3 ± 2.09	a-d	52.3 ± 3.13	a-d	9.5 ± 0.69	ab	6.9 ± 0.41	a-c
**15 ppm AgNPs enc**	20.6 ± 2.66	a-d	52.3 ± 3.52	a-d	8.9 ± 0.56	a-c	6.9 ± 0.57	a-c
**5 ppm AuNPs enc**	25.4 ± 2.63	a	59.4 ± 3.83	a	9.9 ± 0.68	ab	8.1 ± 0.60	a
**15 ppm AuNPs enc**	23.0 ± 2.23	ab	56.4 ± 3.27	a-c	10.0 ± 0.63	a	7.5 ± 0.51	ab
**5 ppm ZnONPs enc**	19.6 ± 2.51	a-d	46.9 ± 4.26	b-e	7.7 ± 0.68	bc	6.4 ± 0.63	b-d
**15 ppm ZnONPs enc**	15.8 ± 2.11	c-e	45.1 ± 3.79	c-e	8.2 ± 0.74	a-c	5.7 ± 0.45	de
	**Valentine**							
**control**	18.3 ± 1.40	a-c	49.7 ± 2.23	a-c	9.5 ± 0.54	ab	6.5 ± 0.28	a-d
**5 ppm AgNPs prec**	19.6 ± 2.05	ab	48.4 ± 3.32	a-d	9.0 ± 0.63	a-c	6.4 ± 0.41	a-d
**15 ppm AgNPs prec**	10.6 ± 1.50	e	34.1 ± 3.31	e	6.8 ± 0.63	cd	4.3 ± 0.37	e
**5 ppm AuNPs prec**	14.6 ± 1.92	b-e	42.2 ± 3.31	b-e	7.9 ± 0.60	b-d	5.2 ± 0.33	c-e
**15 ppm AuNPs prec**	11.4 ± 1.32	de	35.5 ± 2.35	e	6.5 ± 0.47	d	5.0 ± 0.35	de
**5 ppm ZnONPs prec**	17.2 ± 1.69	b-d	47.1 ± 2.81	a-d	8.6 ± 0.56	b-d	6.6 ± 0.47	a-c
**15 ppm ZnONPs prec**	18.0 ± 2.36	a-c	48.9 ± 3.86	a-d	9.2 ± 0.66	ab	6.2 ± 0.47	a-d
**5 ppm AgNPs enc**	23.4 ± 1.30	a	57.6 ± 2.49	a	10.8 ± 0.55	a	7.6 ± 0.38	a
**15 ppm AgNPs enc**	16.6 ± 2.10	b-e	42.7 ± 4.00	b-e	8.1 ± 0.82	b-d	5.9 ± 0.58	b-d
**5 ppm AuNPs enc**	13.2 ± 1.92	c-e	38.5 ± 3.51	de	7.6 ± 0.72	b-d	5.0 ± 0.50	de
**15 ppm AuNPs enc**	14.8 ± 2.79	b-e	40.3 ± 5.07	c-e	7.5 ± 0.94	b-d	5.3 ± 0.59	c-e
**5 ppm ZnONPs enc**	20.5 ± 1.77	ab	52.8 ± 3.03	ab	9.3 ± 0.58	ab	7.4 ± 0.57	ab
**15 ppm ZnONPs enc**	20.8 ± 2.41	ab	51.2 ± 3.45	a-c	8.9 ± 0.63	a-c	7.3 ± 0.61	ab
	**Average width (cm)**		**Aspect ratio**		**Form coefficient**			
**Treatment**	**Gold Heart**							
**control**	2.0 ± 0.16	d	0.77 ± 0.06	a	0.11 ± 0.02		a	
**5 ppm AgNPs prec**	3.6 ± 0.28	a	0.96 ± 0.07	a	0.09 ± 0.00		a	
**15 ppm AgNPs prec**	2.9 ± 0.26	a-c	0.78 ± 0.06	a	0.09 ± 0.01		a	
**5 ppm AuNPs prec**	3.0 ± 0.21	ab	0.84 ± 0.06	a	0.09 ± 0.00		a	
**15 ppm AuNPs prec**	2.8 ± 0.18	b-d	0.76 ± 0.05	a	0.09 ± 0.01		a	
**5 ppm ZnONPs prec**	3.0 ± 0.21	ab	0.71 ± 0.04	a	0.11 ± 0.02		a	
**15 ppm ZnONPs prec**	2.2 ± 0.16	cd	0.80 ± 0.07	a	0.09 ± 0.00		a	
**5 ppm AgNPs enc**	2.7 ± 0.18	bc	0.84 ± 0.10	a	0.09 ± 0.00		a	
**15 ppm AgNPs enc**	2.9 ± 0.28	a-c	0.81 ± 0.05	a	0.09 ± 0.00		a	
**5 ppm AuNPs enc**	3.3 ± 0.27	ab	0.91 ± 0.08	a	0.09 ± 0.00		a	
**15 ppm AuNPs enc**	2.9 ± 0.18	a-c	0.81 ± 0.06	a	0.09 ± 0.00		a	
**5 ppm ZnONPs enc**	2.9 ± 0.30	a-c	0.83 ± 0.07	a	0.10 ± 0.01		a	
**15 ppm ZnONPs enc**	2.2 ± 0.18	cd	0.78 ± 0.07	a	0.09 ± 0.00		a	
	**Valentine**							
**control**	2.4 ± 0.17	a-d	0.74 ± 0.05	a	0.09 ± 0.00		b	
**5 ppm AgNPs prec**	2.6 ± 0.20	a-c	0.75 ± 0.05	a	0.10 ± 0.01		ab	
**15 ppm AgNPs prec**	1.7 ± 0.12	e	0.68 ± 0.04	a	0.12 ± 0.01		a	
**5 ppm AuNPs prec**	2.2 ± 0.17	b-e	0.70 ± 0.04	a	0.10 ± 0.00		ab	
**15 ppm AuNPs prec**	2.1 ± 0.16	c-e	0.83 ± 0.06	a	0.11 ± 0.01		ab	
**5 ppm ZnONPs prec**	2.5 ± 0.22	a-d	0.82 ± 0.07	a	0.09 ± 0.00		b	
**15 ppm ZnONPs prec**	2.3 ± 0.23	a-e	0.68 ± 0.06	a	0.10 ± 0.01		ab	
**5 ppm AgNPs enc**	2.9 ± 0.20	a	0.79 ± 0.07	a	0.09 ± 0.00		b	
**15 ppm AgNPs enc**	2.3 ± 0.23	a-e	0.84 ± 0.08	a	0.12 ± 0.01		a	
**5 ppm AuNPs enc**	1.9 ± 0.20	de	0.72 ± 0.07	a	0.10 ± 0.01		ab	
**15 ppm AuNPs enc**	2.1 ± 0.27	c-e	0.79 ± 0.10	a	0.10 ± 0.01		ab	
**5 ppm ZnONPs enc**	2.7 ± 0.18	a-c	0.86 ± 0.08	a	0.09 ± 0.00		b	
**15 ppm ZnONPs enc**	2.8 ± 0.28	ab	0.87 ± 0.08	a	0.09 ± 0.00		b	

* Each number represents the mean value ± standard error. Significant differences in values are determined by Duncan’s *post hoc* test (*P*<0.05). Values with at least one same letter are not statistically different.

On the other hand, none of the experimental treatments stimulated the development of leaves in cv. ‘Valentine’ compared to the control, although there was a tendency observed suggesting a positive influence of 5 ppm AgNPs in the alginate bead matrix on foliar growth (all of the studied traits reached the highest values in this combination) (Table 2). Conversely, the smallest leaves (in terms of all analyzed parameters) were found in the experimental treatment 15 ppm AgNPs in the preculture medium. No change in the aspect ratio was found, however, leaves of ‘Valentine’ plants from the treatments 15 ppm AgNPs in the preculture medium or alginate bead, with a higher form coefficient value, were more filiform in shape than the control (Table 2).

### Effect of nanoparticles on the physiological condition of LN-derived plants

Plants of *L*. *spectabilis* ‘Gold Heart’ contained significantly less flavonoids and anthocyanins in the leaves if grown on the preculture medium with ZnONPs (regardless of concentration). On the other hand, the same experimental treatments stimulated the synthesis of chlorophyll and provided the highest SPAD value, which was over two-fold higher than in the control (Table 3).

**Table 3:** Effect of silver (AgNPs), gold (AuNPs), and zinc oxide (ZnONPs) nanoparticles applied during the preculture (prec) or encapsulation (enc) step of the encapsulation-vitrification cryopreservation protocol on the relative content of flavonoids, anthocyanins, chlorophyll (CCI) and Leaf Soil-Plant Analysis Development (SPAD) value in the leaves after 70 days of *ex vitro* growth in *Lamprocapnos spectabilis* ‘Gold Heart’ and ‘Valentine’.

	Flavonoids		Anthocyanins		CCI		SPAD	
**Treatment**	**Gold Heart**							
**control**	0.26 ± 0.03	a	0.19 ± 0.02	ab	3.5 ± 0.52	c	13.8 ± 1.76	cd
**5 ppm AgNPs prec**	0.22 ± 0.04	ab	0.21 ± 0.02	ab	2.8 ± 0.22	c	11.4 ± 1.36	d
**15 ppm AgNPs prec**	0.22 ± 0.03	ab	0.22 ± 0.02	a	3.7 ± 0.33	c	15.8 ± 1.30	cd
**5 ppm AuNPs prec**	0.22 ± 0.02	ab	0.22 ± 0.03	a	2.9 ± 0.25	c	12.4 ± 1.23	cd
**15 ppm AuNPs prec**	0.30 ± 0.04	a	0.20 ± 0.02	ab	3.5 ± 0.26	c	15.2 ± 1.12	cd
**5 ppm ZnONPs prec**	0.09 ± 0.02	cd	0.06 ± 0.01	de	8.3 ± 0.81	b	27.4 ± 1.88	b
**15 ppm ZnONPs prec**	0.05 ± 0.01	d	0.02 ± 0.00	e	12.2 ± 0.71	a	35.8 ± 1.00	a
**5 ppm AgNPs enc**	0.15 ± 0.02	bc	0.11 ± 0.02	cd	3.2 ± 0.37	c	13.0 ± 1.97	cd
**15 ppm AgNPs enc**	0.25 ± 0.02	a	0.15 ± 0.02	bc	3.8 ± 0.30	c	16.0 ± 1.43	cd
**5 ppm AuNPs enc**	0.22 ± 0.02	ab	0.20 ± 0.02	ab	3.7 ± 0.30	c	15.8 ± 1.40	cd
**15 ppm AuNPs enc**	0.24 ± 0.03	a	0.19 ± 0.03	ab	4.0 ± 0.38	c	17.3 ± 1.43	c
**5 ppm ZnONPs enc**	0.29 ± 0.03	a	0.25 ± 0.02	ab	3.2 ± 0.28	c	13.7 ± 1.41	cd
**15 ppm ZnONPs enc**	0.25 ± 0.02	a	0.21 ± 0.02	ab	3.2 ± 0.22	c	14.2 ± 1.02	cd
	**Valentine**							
**control**	0.06 ± 0.01	a-c	0.020 ± 0.00	d	10.4 ± 1.24	a-c	31.7 ± 2.37	ab
**5 ppm AgNPs prec**	0.06 ± 0.01	a-c	0.051 ± 0.01	ab	7.9 ± 0.67	bc	26.6 ± 1.79	bc
**15 ppm AgNPs prec**	0.03 ± 0.00	c	0.021 ± 0.00	cd	12.2 ± 1.05	a	34.6 ± 2.39	a
**5 ppm AuNPs prec**	0.05 ± 0.01	a-c	0.027 ± 0.01	b-d	11.3 ± 3.16	ab	30.0 ± 2.45	a-c
**15 ppm AuNPs prec**	0.07 ± 0.01	a-c	0.051 ± 0.01	ab	7.0 ± 0.71	c	24.5 ± 2.08	c
**5 ppm ZnONPs prec**	0.06 ± 0.01	a-c	0.045 ± 0.01	a-c	9.1 ± 0.93	a-c	30.1 ± 1.84	a-c
**15 ppm ZnONPs prec**	0.04 ± 0.01	bc	0.028 ± 0.01	b-d	9.6 ± 0.51	a-c	31.8 ± 0.81	ab
**5 ppm AgNPs enc**	0.06 ± 0.01	a-c	0.030 ± 0.00	a-d	10.9 ± 1.10	a-c	32.4 ± 2.00	ab
**15 ppm AgNPs enc**	0.05 ± 0.01	a-c	0.037 ± 0.01	a-d	9.8 ± 0.83	a-c	31.8 ± 1.54	ab
**5 ppm AuNPs enc**	0.08 ± 0.01	a	0.053 ± 0.01	a	8.3 ± 0.83	a-c	28.5 ± 2.08	a-c
**15 ppm AuNPs enc**	0.07 ± 0.01	ab	0.031 ± 0.01	a-d	8.5 ± 0.67	a-c	29.0 ± 1.97	a-c
**5 ppm ZnONPs enc**	0.04 ± 0.01	bc	0.019 ± 0.00	d	10.2 ± 0.93	a-c	31.8 ± 1.97	ab
**15 ppm ZnONPs enc**	0.05 ± 0.01	a-c	0.025 ± 0.01	cd	11.0 ± 0.61	a-c	34.1 ± 0.89	a

* Each number represents the mean value ± standard error. Significant differences in values are determined by Duncan’s *post hoc* test (*P*<0.05). Values with at least one same letter are not statistically different.

In ‘Valentine’ cultivar, none of the NPs-treated plants had more or less flavonoids or chlorophylls in the leaves than the untreated control (Table 3). Likewise, no increase in the SPAD value was found compared to the control. Conversely, leaves of plants from the treatments 5 ppm AgNPs, 5 ppm ZnONPs or 15 ppm AuNPs in the preculture medium and 5 ppm AuNPs in the alginate bead contained more anthocyanins (Table 3).

As for the relative content of metabolites in ‘Gold Heart’ stems, it was found that augmentation of preculture medium with 15 ppm AuNPs stimulated the synthesis of flavonoids and anthocyanins, whereas the addition of 15 ppm AgNPs into the alginate bead enhanced the synthesis of chlorophyll compared to the control (Table 4).

**Table 4:** Effect of silver (AgNPs), gold (AuNPs), and zinc oxide (ZnONPs) nanoparticles applied during the preculture (prec) or encapsulation (enc) step of the encapsulation-vitrification cryopreservation protocol on the relative content of flavonoids, anthocyanins, and chlorophyll (CCI) in the stems after 70 days of *ex vitro* growth in *Lamprocapnos spectabilis* ‘Gold Heart’ and ‘Valentine’.

	Flavonoids		Anthocyanins		CCI	
**Treatment**	**Gold Heart**					
**control**	0.39 ± 0.06	cd	0.33 ± 0.09	e	0.94 ± 0.02	b
**5 ppm AgNPs prec**	0.42 ± 0.08	cd	0.57 ± 0.12	b-d	1.07 ± 0.02	b
**15 ppm AgNPs prec**	0.37 ± 0.07	cd	0.66 ± 0.08	bc	0.98 ± 0.04	b
**5 ppm AuNPs prec**	0.63 ± 0.11	ab	0.89 ± 0.06	a	0.94 ± 0.07	b
**15 ppm AuNPs prec**	0.68 ± 0.07	a	0.78 ± 0.04	ab	0.93 ± 0.04	b
**5 ppm ZnONPs prec**	0.23 ± 0.04	d	0.45 ± 0.02	c-e	1.01 ± 0.03	b
**15 ppm ZnONPs prec**	0.21 ± 0.04	d	0.33 ± 0.04	e	0.95 ± 0.02	b
**5 ppm AgNPs enc**	0.24 ± 0.09	d	0.40 ± 0.10	de	1.00 ± 0.09	b
**15 ppm AgNPs enc**	0.30 ± 0.09	cd	0.38 ± 0.08	de	1.39 ± 0.35	a
**5 ppm AuNPs enc**	0.33 ± 0.03	cd	0.39 ± 0.06	de	1.11 ± 0.09	ab
**15 ppm AuNPs enc**	0.48 ± 0.08	bc	0.66 ± 0.08	bc	0.92 ± 0.05	b
**5 ppm ZnONPs enc**	0.42 ± 0.06	cd	0.60 ± 0.08	b-d	0.98 ± 0.07	b
**15 ppm ZnONPs enc**	0.38 ± 0.08	cd	0.53 ± 0.07	c-e	0.94 ± 0.02	b
	**Valentine**					
**control**	0.13 ± 0.03	cd	0.25 ± 0.05	de	0.99 ± 0.05	a
**5 ppm AgNPs prec**	0.23 ± 0.03	a-d	0.40 ± 0.04	b-e	1.03 ± 0.05	a
**15 ppm AgNPs prec**	0.26 ± 0.04	a-c	0.41 ± 0.03	b-e	0.95 ± 0.04	a
**5 ppm AuNPs prec**	0.31 ± 0.03	ab	0.48 ± 0.01	b	0.90 ± 0.05	a
**15 ppm AuNPs prec**	0.23 ± 0.05	a-d	0.42 ± 0.04	b-d	0.93 ± 0.03	a
**5 ppm ZnONPs prec**	0.28 ± 0.04	a-c	0.44 ± 0.04	bc	0.85 ± 0.04	a
**15 ppm ZnONPs prec**	0.21 ± 0.03	a-d	0.48 ± 0.02	b	0.97 ± 0.05	a
**5 ppm AgNPs enc**	0.34 ± 0.09	a	0.64 ± 0.10	a	0.99 ± 0.03	a
**15 ppm AgNPs enc**	0.19 ± 0.04	a-d	0.33 ± 0.07	b-e	0.98 ± 0.04	a
**5 ppm AuNPs enc**	0.18 ± 0.05	b-d	0.29 ± 0.08	c-e	1.04 ± 0.05	a
**15 ppm AuNPs enc**	0.14 ± 0.04	cd	0.26 ± 0.06	c-e	0.98 ± 0.03	a
**5 ppm ZnONPs enc**	0.20 ± 0.04	a-d	0.24 ± 0.05	e	0.97 ± 0.02	a
**15 ppm ZnONPs enc**	0.09 ± 0.02	d	0.29 ± 0.03	c-e	1.02 ± 0.03	a

* Each number represents the mean value ± standard error. Significant differences in values are determined by Duncan’s *post hoc* test (*P*<0.05). Values with at least one same letter are not statistically different.

On the other hand, 5 ppm AuNPs and AgNPs in the preculture medium and alginate bead, respectively, stimulated the production of flavonoids and anthocyanins in the stems of *L*. *spectabilis* ‘Valentine’. Moreover, the concentration of anthocyanins was increased by the presence of 15 ppm ZnONPs in the preculture medium compared to the untreated control (Table 4). No effect of the applied nanoparticles was found in terms of chlorophyll content in this cultivar.

The application of ZnONPs during the preculture step increased the chlorophyll fluorescence parameters (F0, Fv, and Fm) in ‘Gold Heart’ cultivar, although the Fv/Fm and Fv/F0 values remained unchanged compared to the control. Also in ‘Valentine’ the presence of 15 ppm ZnONPs in the preculture medium increased the initial and maximal chlorophyll fluorescence but the Fv/Fm and Fv/F0 values were the same in all experimental treatments (Table 5).

**Table 5:** Effect of silver (AgNPs), gold (AuNPs), and zinc oxide (ZnONPs) nanoparticles applied during the preculture (prec) or encapsulation (enc) step of the encapsulation-vitrification cryopreservation protocol on the chlorophyll fluorescence parameters and ratios in the leaves after 70 days of *ex vitro* growth in *Lamprocapnos spectabilis* ‘Gold Heart’ and ‘Valentine’.

	F0		Fv		Fm		Fv/Fm		Fv/F0	
**Treatment**	**Gold Heart**									
**control**	108.0 ± 9.6	c	492.5 ± 45.7	c	600.5 ± 52.8	c	0.82 ± 0.01	ab	4.6 ± 0.29	a-c
**5 ppm AgNPs prec**	99.8 ± 11.6	c	448.3 ± 46.5	c	548.0 ± 58.0	c	0.82 ± 0.00	ab	4.5 ± 0.07	a-c
**15 ppm AgNPs prec**	122.7 ± 18.7	c	537.5 ± 75.5	c	660.2 ± 92.7	c	0.81 ± 0.01	ab	4.4 ± 0.27	a-c
**5 ppm AuNPs prec**	82.8 ± 14.1	c	353.0 ± 56.1	c	435.8 ± 68.0	c	0.81 ± 0.01	ab	4.4 ± 0.36	a-c
**15 ppm AuNPs prec**	89.0 ± 8.8	c	405.1 ± 45.4	c	494.1 ± 53.3	c	0.82 ± 0.01	ab	4.6 ± 0.27	a-c
**5 ppm ZnONPs prec**	206.1 ± 20.8	b	783.3 ± 66.4	b	989.4 ± 84.9	b	0.79 ± 0.01	b	3.9 ± 0.28	c
**15 ppm ZnONPs prec**	286.8 ± 12.2	a	1112.8 ± 68.9	a	1399.5 ± 76.0	a	0.79 ± 0.01	b	3.9 ± 0.22	c
**5 ppm AgNPs enc**	140.3 ± 32.5	c	577.5 ± 115.2	c	717.8 ± 147.4	c	0.81 ± 0.01	ab	4.3 ± 0.20	bc
**15 ppm AgNPs enc**	124.2 ± 25.1	c	533.2 ± 80.4	c	657.4 ± 105.2	c	0.82 ± 0.01	ab	4.5 ± 0.23	a-c
**5 ppm AuNPs enc**	96.3 ± 12.5	c	487.4 ± 62.3	c	583.7 ± 74.7	c	0.83 ± 0.00	a	5.1 ± 0.13	ab
**15 ppm AuNPs enc**	87.3 ± 11.8	c	450.0 ± 53.1	c	537.3 ± 64.8	c	0.84 ± 0.00	a	5.2 ± 0.18	a
**5 ppm ZnONPs enc**	102.8 ± 16.7	c	443.5 ± 34.0	c	546.3 ± 49.8	c	0.82 ± 0.01	ab	4.6 ± 0.40	a-c
**15 ppm ZnONPs enc**	82.0 ± 9.2	c	361.9 ± 42.8	c	443.9 ± 51.2	c	0.81 ± 0.01	ab	4.4 ± 0.21	a-c
	**Valentine**									
**control**	230.1 ± 13.9	bc	797.0 ± 65.9	ab	1027.1 ± 79.0	bc	0.77 ± 0.01	a	3.4 ± 0.15	a
**5 ppm AgNPs prec**	264.6 ± 18.9	b	843.7 ± 40.0	ab	1108.2 ± 58.0	a-c	0.76 ± 0.01	a	3.2 ± 0.12	a
**15 ppm AgNPs prec**	256.5 ± 12.1	b	959.7 ± 53.4	a	1216.2 ± 65.1	ab	0.79 ± 0.00	a	3.7 ± 0.07	a
**5 ppm AuNPs prec**	224.0 ± 23.7	bc	732.3 ± 81.8	b	956.3 ± 103.9	c	0.76 ± 0.01	a	3.3 ± 0.15	a
**15 ppm AuNPs prec**	247.9 ± 8.2	bc	818.5 ± 20.6	ab	1066.4 ± 25.2	a-c	0.77 ± 0.01	a	3.3 ± 0.11	a
**5 ppm ZnONPs prec**	278.7 ± 12.6	ab	955.8 ± 52.7	a	1234.5 ± 60.2	ab	0.77 ± 0.01	a	3.4 ± 0.17	a
**15 ppm ZnONPs prec**	328.7 ± 20.6	a	938.3 ± 53.5	a	1267.0 ± 67.1	a	0.74 ± 0.01	a	2.9 ± 0.15	a
**5 ppm AgNPs enc**	244.0 ± 13.1	bc	782.1 ± 43.3	ab	1026.1 ± 47.0	bc	0.76 ± 0.01	a	3.3 ± 0.23	a
**15 ppm AgNPs enc**	239.5 ± 25.2	bc	732.0 ± 44.1	b	971.5 ± 52.1	c	0.75 ± 0.02	a	3.2 ± 0.35	a
**5 ppm AuNPs enc**	192.0 ± 34.1	c	680.0 ± 114.3	b	872.0 ± 147.5	c	0.78 ± 0.01	a	3.6 ± 0.15	a
**15 ppm AuNPs enc**	282.4 ± 23.0	ab	803.8 ± 64.4	ab	1086.2 ± 82.7	a-c	0.74 ± 0.01	a	2.9 ± 0.17	a
**5 ppm ZnONPs enc**	251.3 ± 9.3	b	804.0 ± 47.6	ab	1055.3 ± 47.3	a-c	0.76 ± 0.01	a	3.2 ± 0.22	a
**15 ppm ZnONPs enc**	233.3 ± 6.8	bc	771.3 ± 40.5	ab	1004.6 ± 36.8	bc	0.76 ± 0.01	a	3.3 ± 0.23	a

* Each number represents the mean value ± standard error. Significant differences in values are determined by Duncan’s *post hoc* test (*P*<0.05). Values with at least one same letter are not statistically different.

### Effect of nanoparticles on the nuclear DNA content in LN-derived plants

**Fig 1:**
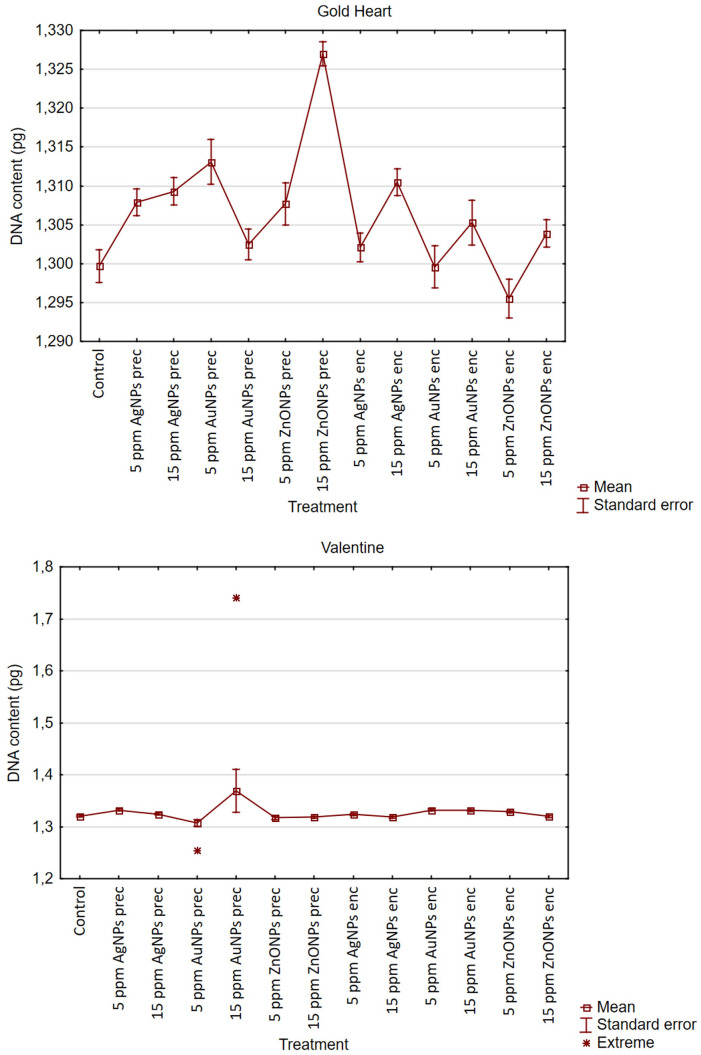
Effect of silver (AgNPs), gold (AuNPs), and zinc oxide (ZnONPs) nanoparticles applied during the preculture (prec) or encapsulation (enc) step of the encapsulation-vitrification cryopreservation protocol on the DNA content in the shoots of *ex vitro*-grown *Lamprocapnos spectabilis* ‘Gold Heart’ and ‘Valentine’.

**Fig 2:**
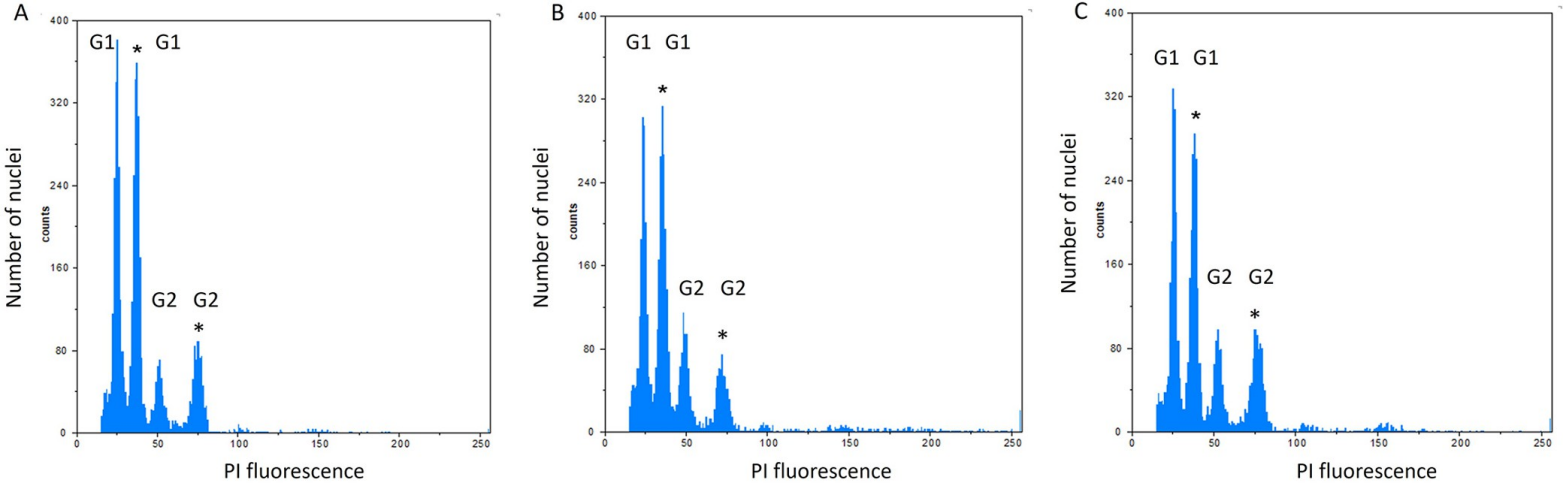
Example FCM histograms of fluorescence intensity of propidium iodide in the nuclei of *Lamprocapnos spectabilis* ‘Valentine’ plants: A–control; B– 5 ppm AuNPs in the preculture medium; C– 15 ppm AuNPs in the preculture medium. The peaks of the internal standard (*Solanum lycopersicum* ´Stupicke’) are indicated with an asterisk (*).

Supplementation of the preculture medium with 15 ppm ZnONPs increased the nuclear DNA content in the ‘Gold Heart’ plants (mean 1.327 pg) compared to all other treatments and the control (1.296–1.3131 pg) (Fig 1). As for the ‘Valentine’ cultivar, two specimens from the treatments 5 and 15 ppm AuNPs in the preculture medium, had a significantly different DNA content than the remaining samples. One of these specimens displayed a notably reduced DNA content (1.254 pg), whereas the other exhibited an elevated DNA content (1.741 pg; Fig 2).

## Discussion

Nanoparticles, with their small size and unique properties, offer novel solutions to some of the longstanding challenges in cryobiology. In this study, we focused on nanoparticle-assisted cryopreservation, to explore the multifaceted ways in which nanoparticles can improve the preservation of plant specimens.

### Effect of nanoparticles on the survival and biometric parameters of *L*. *spectabilis* plants *ex vitro*

The use of NPs in agriculture and horticulture can be considered a double-edged sword. It is known that nanoparticles can benefit or harm plants, based on NPs type, concentration and exposure time [43]. The lack of impact on the survival and leaf number in *L*. *spectabilis* ’Gold Heart’ during acclimatization may indicate a higher tolerance of this cultivar to the tested nanoparticles. On the other hand, the significant increase in shoot length when supplementing alginate with 5 ppm AuNPs suggests a potential positive role of gold nanoparticles in promoting shoot elongation. Likewise, gold-nanoparticle enhanced the growth and seed yield of *Brassica juncea* (L.) Czern. [44]. Differential transcriptomic and proteomic analyses in *Arabidopsis* demonstrated a downregulation in oxidative stress responses while growth-promoting genes/proteins were upregulated as a result of AuNPs treatment [32], which coincides with our observations. The growth-stimulating properties of nanoparticles could also explain why most of the treatments (usually except for ZnONPs) had a positive effect on the leaf development in the ‘Gold Heart’ cultivar. Silica nanoparticles (SiNPs) also had beneficial effects on the growth of leaves and stems in wheat (*Triticum aestivum* L.) [45]. Nanoparticles can stimulate foliar growth and development partly by regulating the metabolisms and synthesis of plant hormones [46]. The observed acceleration in plant growth is highly beneficial and confirms the utility of nanoparticles in the cryopreservation of this cultivar. Since the best results were obtained with a concentration of 5 ppm NPs, it is recommended to use lower doses of nanoparticles when supplementing the preculture medium or alginate beads. This is in agreement with the findings of other authors, who also described that lower concentrations of gold nanoparticles (10 ppm) had a positive effect on the survival of LN-derived shoot tips in *L*. *spectabilis* [20].

*L*. *spectabilis* ’Valentine’ exhibited a more varied response in the present study. The stimulation of shoot elongation with 5 ppm AgNPs or 5 ppm ZnONPs in the alginate bead matrix suggests that these NPs might have growth-promoting effects in this cultivar. Nanoparticles regulate phytohormone biosynthesis and signaling in plants [47]. They can be used as carrier systems of auxins and gibberellins [48], which could explain the observed here results. On the other hand, the adverse impact on the acclimatization efficiency with 15 ppm ZnONPs in the preculture medium could be indicative of increased sensitivity to this particular nanoparticle concentration. Cytotoxic effects of NPs have been reported in several plant studies [49]. ZnONPs exhibited phytotoxic effects on soybean plantlets at elevated concentrations [50]. Copper oxide nanoparticles (CuONPs), in general, had a positive or neutral effect on root and shoot growth by enhancing photosynthesis and nutrient uptake in several monocots and dicots. However, high concentrations of CuONPs caused oxidative stress and damage to plant cells, resulting in reduced growth and yield [43]. Likewise, in *L*. *spectabilis* ’Valentine’, the presence of a higher concentration of silver and gold nanoparticles in the preculture medium resulted in the development of smaller leaves. In contrast, a tendency with 5 ppm AgNPs in the alginate bead matrix suggests a potential positive influence on foliar growth.

The observed varied effects of NPs on *L*. *spectabilis* ’Gold Heart’ and ’Valentine’ can be attributed to the distinct characteristics and responses of each cultivar to the nanoparticle treatments. Not many studies though focus on the effect of various types of nanoparticles on different plant cultivars. Nevertheless, seed priming using selenium nanoparticles (SeNPs) resulted in varied chemical and biological properties of three *Brassica oleracea* cultivars [51]. Likewise, the application of AgNPs did not affect the germination efficiency; however, diverse results were reported for the growth and biochemical activity in the seedling of three vegetable species: *Solanum lycopersicum* L., *Raphanus sativus* L. var. *sativus* and *Brassica oleracea* var. *sabellica* [52].

### Effect of nanoparticles on the physiological state of *L*. *spectabilis* plants

Nanoparticle treatments altered the physiological response of LN-derived specimens. NPs are known to affect the biochemical profile of plants [53]. Surprisingly, in the present study, this effect depended not only on the cultivar but also on the plant organ (different responses of leaves and stems). This phenomenon can be explained as leaves and stems serve different functions in plants and have distinct metabolic activities. Moreover, it is known that nanoparticles can accumulate in plants although the mechanism of such accumulation and the route of the movement of nanoparticles in various organs have not yet been fully explained [54]. According to previous research, the same kind of nanoparticle differs in translocation and accumulation in different plant species but also within the same plant [55, 56]. The obtained here results suggest that the specific mechanisms of nanoparticle-plant interaction are complex and may involve interactions at the genetic, enzymatic, and signaling levels.

The differences in chlorophyll fluorescence and photosynthetic apparatus activity further highlight the specificity of the nanoparticle effects. Leaf Soil-Plant Analysis Development (SPAD) prediction is a crucial measure of plant health. SPAD value is correlated with the amount of nitrogen (an important plant nutrient) present in the leaf [57]. A higher SPAD value indicates a healthier plant. Therefore, it can be assumed that the utilization of ZnONPs in the cryopreservation procedure may be highly beneficial. This hypothesis is supported by the accompanying increased activity of PSII and decline in anthocyanin content as anthocyanins are considered an oxidative stress marker [58].

Most metallic nanoparticles are harmful to the photosynthetic apparatus by causing both structural and functional damage and disrupting redox homeostasis in cells [59]. On the other hand, it was found that TiO_2_NPs may enhance the efficiency of photosynthesis. In the present study, no negative impact of nanoparticles on the stress indicators (Fv/Fm and F0/Fv) was found compared to the control, confirming the utility of NPs in cryobiology research. It is worth noticing that in the ‘Gold Heart’ plants, the Fv/Fm values were in the range of 0.79–0.84, which is the optimal value for most plant species [60]. However, in ‘Valentine’ only the plants from the treatment 15 ppm AgNPs in the preculture medium reached the “safe” 0.79 value. All other combinations, including the control, were in the range of 0.74–0.78 indicating that this cultivar is generally more stress-susceptible than ‘Gold Heart’. The reason why the former cultivar is less stress-tolerant could be attributed to the lower concentration of plant pigments (flavonoids and anthocyanins), which play a significant function in the plant’s defense mechanism [58]. This could also explain why some of the treatments with higher concentrations of NPs caused a decline in the acclimatization efficiency (60% survival) of ‘Valentine’ plants.

### Effect of nanoparticles on the cytogenetic events in *L*. *spectabilis* plants

Cryopreservation is considered an ideal strategy for the long-term preservation of plant germplasm, particularly in terms of maintaining its genetic stability [22]. Usually, the 2C DNA content and ploidy level of cryopreserved plants are the same as in the untreated control [61], although sometimes cryostorage may affect the cell cycle. According to the previous research, cell nuclei after cryopreservation are mainly in the G1/G0 phase (DNA content at the 2C level), whereas fewer cells are in the G2 phase [62], which corresponds with the results obtained in the current research. Nonetheless, nanoparticles may affect the DNA synthesis in the plant cells. Due to their small size, NPs are easily absorbed by the cell, even penetrating the caryolemma, and interacting directly or indirectly with DNA or the mechanisms of its synthesis, methylation, reparation *etc*. [63]. Nanoparticles can cause various types of structural chromosomal aberrations, such as incorrect orientation at metaphase, chromosomal breakage, metaphasic plate distortion, spindle dysfunction, stickiness, aberrant movement at metaphase, fragmentation, scattering, unequal separation, chromosomal gaps, multipolar anaphase, erosion, as well as distributed and lagging chromosomes, micronuclei induction, and decrease the value of the mitotic index [64, 65].

In the present study, it was found that one of the ‘Valentine’ specimens obtained from the experimental treatment 5 ppm AuNPs in the preculture medium contained significantly less DNA (1.254 pg) than the remaining plants from the same treatment (1.324 pg). This can be explained by the findings of other authors, who claim that NPs may inhibit DNA replication by binding to DNA [66]. On the other hand, another specimen from the treatment 15 ppm AuNPs in the preculture medium contained a much higher nuclear DNA content (1.741 pg). This could result from an altered mitotic index and explain why gold nanoparticles stimulated the growth of *L*. *spectabilis in vitro* when added to the culture medium at higher concentration [67]. A similar phenomenon was reported with cadmium nanoparticles (CdNPs) and *Eruca sativa* Mill. [68], as well as silver nanoparticles and *Allium* plants [69]. Nonetheless, one should keep in mind that the severity of NPs-induced abnormalities depends on the type, concentration, exposition time and particle size [70]. This could explain why a higher (15 ppm) concentration of bigger (13 nm) ZnONPs caused more evident changes in the DNA content of ‘Gold Heart’ plants (1.327 pg) when added at the preculture step for an entire week before cryopreservation compared to the smaller (6 nm) gold or silver nanoparticles or even ZnONPs that were used at lower doses (5 ppm) or added into the alginate bead matrix directly before LN-storage (1.296–1.313 pg). Zinc has a structural function in proteins that are involved in DNA replication [71] and stimulates DNA synthesis [72]. The observed increase in DNA synthesis and nuclear activity could explain the elevation of chlorophyll content and SPAD index value in leaves. Further chromosome analyses, e.g. fluorescence *in situ* hybridization (FISH), would be necessary to detect the precise mechanism of NPs action. Nonetheless, the observed difference in DNA content is, in most cases, very small (~0.03 pg 2C^-1^) and does not affect the ploidy level of the plants. Minor changes in the number of chromosomes of LN-derived *Fragaria × ananasa* Duchesne plants were observed by [73], while [74] detected genome size changes (at the level ~0.01 pg 2C^-1^) in cryopreserved somatic embryos of *Quercus suber* L. that were all considered irrelevant.

## Conclusions

The interdisciplinary fusion of nanotechnology and cryobiology holds great promise for addressing global challenges in biodiversity security and sustainability. In this study, the utility of nanoparticles in the cryopreservation of *L*. *spectabilis* was verified.

The study revealed that the influence of nanoparticles on plant responses is rather complex. *L*. *spectabilis* ’Gold Heart’ exhibited a generally positive response, with nanoparticles enhancing plant survival, shoot length, and leaf development. The varied effects observed in ’Valentine’ cultivar, such as stimulation of shoot elongation with specific nanoparticle treatments, suggest that cultivars respond differently to the same nanoparticle treatment. Physiologically, nanoparticles affected the biochemical profile, chlorophyll fluorescence, and photosynthetic apparatus activity. The intricate responses observed in leaves and stems further emphasized the diverse impacts of nanoparticles on different plant organs. Nonetheless, the study suggests that nanoparticles may regulate plant growth and development by influencing its metabolisms. With minimal changes in DNA content observed, the present study highlights the need for further exploration into the long-term effects of nanoparticle exposure on genetic stability in cryopreserved plants.

## Supporting information

S1 Fig *Post*-cryostorage recovery and *ex vitro* growth of *Lamprocapnos spectabilis* ‘Valentine’. A–developing shoot breaking the alginate capsule; B–*in vitro*-recovered plantlets; C–acclimatization in a multipot; D–vegetative growth of plants in a glasshouse; E–measurement of pigment content in leaves; F–flowering plant with no signs of phenotype variation.
(JPG)

## References

[1] Sanzari I, Leone A, Ambrosone A. Nanotechnology in plant science: To make a long story short. Front Bioeng Biotechnol. 2019;7: 120. doi: 10.3389/fbioe.2019.00120 31192203 PMC6550098

[2] Zhao L, Lu L, Wang A, Zhang H, Huang M, Wu H, et al. Nano-biotechnology in agriculture: Use of nanomaterials to promote plant growth and stress tolerance. J Agric Food Chem. 2020;68(7): 1935–1947. doi: 10.1021/acs.jafc.9b06615 32003987

[3] Singh Y, Kumar U, Panigrahi S, Balyan P, Mehla S, Sihag P, et al. Nanoparticles as novel elicitors in plant tissue culture applications: Current status and future outlook. Plant Physiol Biochem. 2023;203: 108004. doi: 10.1016/j.plaphy.2023.108004 37714027

[4] Tymoszuk A, Miler N. Silver and gold nanoparticles impact on *in vitro* adventitious organogenesis in chrysanthemum, gerbera and Cape Primrose. Sci Hortic. 2019;257: 108766. doi: 10.1016/j.scienta.2019.108766

[5] Wu K, Xu C, Li T, Ma H, Gong J, Li X, et al. Application of nanotechnology in plant genetic engineering. Int J Mol Sci. 2023;24(19): 14836. doi: 10.3390/ijms241914836 37834283 PMC10573821

[6] Joshi S, Dar AI, Acharya A, Joshi R. Charged gold nanoparticles promote in vitro proliferation in *Nardostachys jatamansi* by differentially regulating chlorophyll content, hormone concentration, and antioxidant activity. Antioxidants. 2022;11:1962. doi: 10.3390/antiox11101962 36290684 PMC9598260

[7] Polivanova OB, Bedarev VA. Hyperhydricity in plant tissue culture. Plants. 2022;11(23): 3313. doi: 10.3390/plants11233313 36501352 PMC9738826

[8] Tymoszuk A, Wenda-Piesik A, Szałaj U, Wojnarowicz J. Analysis of architecture of chrysanthemum plantlets in response to zinc oxide, silver and auxin treatment in shoot-tip culture. Acta Societatis Botanicorum Poloniae. 2024;93:1–25. doi: 10.5586/asbp/183092

[9] Tymoszuk A, Sławkowska N, Szałaj U, Kulus D, Antkowiak M, Wojnarowicz J. Synthesis, characteristics, and effect of zinc oxide and silver nanoparticles on the in vitro regeneration and biochemical profile of chrysanthemum adventitious shoots. Materials 2022;15:8192. doi: 10.3390/ma15228192 36431675 PMC9696543

[10] Tymoszuk A, Kulus D. Effect of silver nanoparticles on the in vitro regeneration, biochemical, genetic, and phenotype variation in adventitious shoots produced from leaf explants in chrysanthemum. Int J Mol Sci. 2022 Jul 3;23(13): 7406. doi: 10.3390/ijms23137406 35806413 PMC9266331

[11] El Merzougui S, Benelli C, El Boullani R, Serghini MA. The cryopreservation of medicinal and ornamental geophytes: Application and challenges. Plants. 2023;12(11): 2143. doi: 10.3390/plants12112143 37299120 PMC10255897

[12] Benelli C. Plant cryopreservation: A look at the present and the future. Plants. 2021;10(12): 2744. doi: 10.3390/plants10122744 34961214 PMC8707037

[13] Stewart S, Arminan A, He X. Nanoparticle-mediated delivery of cryoprotectants for cryopreservation. Cryo Letters. 2020;41(6): 308–316. doi: 10.1038/npre.2009.3722.1 33814648 PMC8015346

[14] Saadeldin IM, Khalil WA, Alharbi MG, Lee SH. The current trends in using nanoparticles, liposomes, and exosomes for semen cryopreservation. Animals. 2020;10: 2281. doi: 10.3390/ani10122281 33287256 PMC7761754

[15] Hozyen HF, El Shamy AA, Abd El Fattah EM, Sakr AM. Facile fabrication of zinc oxide nanoparticles for enhanced buffalo sperm parameters during cryopreservation. JTEMIN. 2023;4: 100058. doi: 10.1016/j.jtemin.2023.100058

[16] Demirer GS, Zhang H, Goh NS, Pinals RL, Chang R, Landry MP. Carbon nanocarriers deliver siRNA to intact plant cells for efficient gene knockdown. Sci Adv. 2020;6: eaaz0495. doi: 10.1126/sciadv.aaz0495 32637592 PMC7314522

[17] Hosseinmardi M, Siadat F, Sharafi M, Roodbari NH, Hezavehei M. Protective effect of cerium oxide nanoparticles on human sperm function during cryopreservation. Biopreserv Biobank. 2022;20(1): 24–30. doi: 10.1089/bio.2021.0020 34271833

[18] Asadi Z, Safari-Faramani R, Aghaz F. Effects of adding antioxidant nanoparticles on sperm parameters of non-human species after the freezing and thawing process: A systematic review and meta-analysis. Animal Reproduction Science. 2023;257: 107323. doi: 10.1016/j.anireprosci.2023.107323 37666048

[19] Choi HW, Jang H. Application of nanoparticles and melatonin for cryopreservation of gametes and embryos. Curr Issues Mol Biol. 2022;44(9): 4028–4044. doi: 10.3390/cimb44090276 36135188 PMC9497981

[20] Kulus D, Tymoszuk A. Gold nanoparticles affect the cryopreservation efficiency of *in vitro*‑derived shoot tips of bleeding heart. PCTOC. 2021;146: 297–311. doi: 10.1007/s11240-021-02069-4

[21] Kulus D, Tymoszuk A, Kulpińska A, Wojnarowicz J, Szałaj U. Nanoparticle-mediated enhancement of plant cryopreservation: Cultivar-specific insights into morphogenesis and biochemical responses in *Lamprocapnos spectabilis* (L.) Fukuhara ‘Gold Heart’ and ‘Valentine’. PLoS ONE. 2024;19(5): e0304586. doi: 10.1371/journal.pone.0304586 38820507 PMC11142695

[22] Wang MR, Bi W, Shukla MR, Ren L, Hamborg Z, Blystad DR, et al. Epigenetic and genetic integrity, metabolic stability, and field performance of cryopreserved plants. Plants. 2021;10(9): 1889. doi: 10.3390/plants10091889 34579422 PMC8467502

[23] Mosa KA, Ahmed AE, Hazem Y, Kanawati IS, Abdullah A, Hernandez-Sori L, et al. Insights into cryopreservation, recovery and genetic stability of medicinal plant tissues. Fitoterapia. 2023;169: 105555. doi: 10.1016/j.fitote.2023.105555 37295757

[24] Arguedas M, Perez A, Abdelnour A, Hernandez M, Engelmann F, Martínez ME, et al. Short-term liquid nitrogen storage of maize, common bean and soybean seeds modifies their biochemical composition. Agric Sci. 2016;4(3): 6–12. doi: 10.12735/as.v4n3p06

[25] Arguedas M, Gómez D, Hernández L, Engelmann F, Garramone R, Cejas I, et al. Maize seed cryo-storage modifies chlorophyll, carotenoid, protein, aldehyde and phenolics levels during early stages of germination. Acta Physiol Plant. 2018; 40: 118. doi: 10.1007/s11738-018-2695-7

[26] Cejas I, Rumlow A, Turcios A, Engelmann F, Martínez ME, Yabor L, et al. Exposure of common bean seeds to liquid nitrogen modifies mineral composition of young plantlet leaves. Am J Plant Sci. 2016;7(12): 1612–1617. doi: 10.4236/ajps.2016.712152

[27] Zhang Z, Skjeseth G, Elameen A, Haugslien S, Sivertsen A, Wang QC, et al. Field performance evaluation and genetic integrity assessment in *Argyranthemum maderense* plants recovered from cryopreserved shoot tips. In Vitro Cell Dev Biol–Plant. 2015;51: 505–513. doi: 10.1007/s11627-015-9707-8

[28] Kulus D, Rewers M, Serocka M., Mikuła A. Cryopreservation by encapsulation-dehydration affects the vegetative growth of chrysanthemum but does not disturb its chimeric structure. PCTOC. 2019;138(1): 153–166. doi: 10.1007/s11240-019-01614-6

[29] Gao M, Chang J, Wang Z, Zhang H. Advances in transport and toxicity of nanoparticles in plants. J Nanobiotechnol. 2023;21: 75. doi: 10.1186/s12951-023-01830-5 36864504 PMC9983278

[30] Rossi L, Fedenia LN, Sharifan H, Ma X, Lombardini L. Effects of foliar application of zinc sulfate and zinc nanoparticles in coffee (*Coffea arabica* L) plants. Plant Physiol Biochem. 2019;135: 160–6. doi: 10.1016/j.plaphy.2018.12.005 30553137

[31] Yuan J, Chen Y, Li H, Lu J, Zhao H, Liu M, et al. New insights into the cellular responses to iron nanoparticles in *Capsicum annuum*. Sci Rep. 2018;1(8): 3228. doi: 10.1038/s41598-017-18055-w 29459620 PMC5818496

[32] Ferrari E, Barbero F, Busquets-Fité M, Franz-Wachtel M, Köhler HR, Puntes V, et al. Growth-promoting gold nanoparticles decrease stress responses in *Arabidopsis* seedlings. Nanomaterials. 2021;11(12):3161. doi: 10.3390/nano11123161 34947510 PMC8707008

[33] Poddar K, Sarkar D, Sarkar A. Nanoparticles on photosynthesis of plants: Effects and role. In: Patra J, Fraceto L, Das G, Campos E, editors. Green Nanoparticles. Nanotechnology in the Life Sciences. Springer, Cham; 2020. pp. 272–287.

[34] Al-Khayri JM, Rashmi R, Surya Ulhas R, Sudheer WN, Banadka A, Nagella P, et al. The Role of nanoparticles in response of plants to abiotic stress at physiological, biochemical, and molecular levels. Plants. 2023;12(2): 292. doi: 10.3390/plants12020292 36679005 PMC9865530

[35] Nair PMG, Chung IM. Regulation of morphological, molecular and nutrient status in *Arabidopsis thaliana* seedlings in response to ZnO nanoparticles and Zn ion exposure. Sci Total Environ. 2017;575: 187–98. doi: 10.1016/j.scitotenv.2016.10.017 27741454

[36] Tripathi A, Liu S, Singh PK, Kumar N, Pandey AC, Tripathi DK, et al. Differential phytotoxic responses of silver nitrate (AgNO_3_) and silver nanoparticle (Ag NPs) in *Cucumis sativus* L. Plant Gene. 2017;11: 255–264. doi: 10.1016/j.plgene.2017.07.005

[37] Misiurek J, Plech T, Kaproń B, Makuch-Kocka A, Szultka-Młyńska M, Buszewski B, et al. Determination of some isoquinoline alkaloids in extracts obtained from selected plants of the Ranunculaceae, Papaveraceae and Fumarioideae families by liquid chromatography and *in vitro* and *in vivo* investigations of their cytotoxic activity. Molecules 2023;28: 3503. doi: 10.3390/molecules28083503 37110737 PMC10143472

[38] Kulus D. Shoot tip cryopreservation of *Lamprocapnos spectabilis* (L.) Fukuhara using different approaches and evaluation of stability on the molecular, biochemical, and plant architecture levels. Int J Mol Sci. 2020;21(11): 3901. doi: 10.3390/ijms21113901 32486149 PMC7311993

[39] Murashige T, Skoog F. A revised medium for rapid growth and bio assays with tobacco tissue cultures. Physiol Plant. 1962;15: 473–497. doi: 10.1111/j.1399-3054.1962.tb08052.x

[40] RHSCC. The Royal Horticultural Society Colour Chart, London. 1966.

[41] Dolezel J, Greilhuber J, Suda J. Estimation of nuclear DNA content in plants using flow cytometry. Nat Protoc. 2007;2: 2233–2244. doi: 10.1038/nprot.2007.310 17853881

[42] Dolezel J, Sgorbati S, Lucretti S. Comparison of three DNA fluorochromes for flow cytometric estimation of nuclear DNA content in plants. Physiol Plant. 1992;85: 625–631. doi: 10.1111/j.1399-3054.1992.tb04764.x

[43] Feigl G. The impact of copper oxide nanoparticles on plant growth: a comprehensive review. J Plant Interact. 2023;18: 1. doi: 10.1080/17429145.2023.2243098

[44] Arora S, Sharma P, Kumar S, Nayan R, Khanna PK, Zaidi MGH. Gold-nanoparticle induced enhancement in growth and seed yield of *Brassica juncea*. Plant Growth Regul. 2012;66: 303–310. doi: 10.1007/s10725-011-9649-z

[45] Li Y, Xi K, Liu X, Han S, Han X, Li G, et al. Silica nanoparticles promote wheat growth by mediating hormones and sugar metabolism. J Nanobiotechnol. 2023;21(1): 2. doi: 10.1186/s12951-022-01753-7 36593514 PMC9808955

[46] Rasheed A, Li H, Tahir MM, Mahmood A, Nawaz M, Shah AN, et al. The role of nanoparticles in plant biochemical, physiological, and molecular responses under drought stress: A review. Front Plant Sci. 2022;13: 976179. doi: 10.3389/fpls.2022.976179 36507430 PMC9730289

[47] Tripathi D, Singh M, Pandey-Rai S. Crosstalk of nanoparticles and phytohormones regulate plant growth and metabolism under abiotic and biotic stress. Plant Stress. 2022;6: 100107. doi: 10.1016/j.stress.2022.100107

[48] Kokina I, Jahundoviča I, Mickeviča I, Jermaļonoka M, Strautiņš J, Popovs S, et al. Target transportation of auxin on mesoporous Au/SiO_2_ nanoparticles as a method for somaclonal variation increasing in flax (*L*. *usitatissimum* L.). J Nanomater. 2017;vol. 2017: Article ID 7143269, 9 pages. doi: 10.1155/2017/7143269

[49] Tarrahi R, Mahjouri S, Khataee A. A review on *in vivo* and *in vitro* nanotoxicological studies in plants: A headlight for future targets. Ecotoxicol Environ Saf. 2021;208: 111697. doi: 10.1016/j.ecoenv.2020.111697 33396028

[50] Leopold LF, Coman C, Clapa D, Oprea I, Toma A, Iancu ȘD, et al. The effect of 100–200 nm ZnO and TiO2 nanoparticles on the in vitro-grown soybean plants. Colloids Surf B: Biointerfaces. 2022;Volume 216: 112536. doi: 10.1016/j.colsurfb.2022.112536 35567806

[51] AbdElgawad H, Magdy Korany S, Reyad AM, Zahid I, Akhter N, Alsherif E, et al. Synergistic impacts of plant-growth-promoting bacteria and selenium nanoparticles on improving the nutritional value and biological activities of three cultivars of *Brassica* sprouts. ACS Omega. 2023;8(29): 26414–26424. doi: 10.1021/acsomega.3c02957 37521602 PMC10373182

[52] Tymoszuk A. Silver nanoparticles effects on *in vitro* germination, growth, and biochemical activity of tomato, radish, and kale seedlings. Materials. 2021;14(18): 5340. doi: 10.3390/ma14185340 34576564 PMC8468885

[53] Hashemi S, Asrar Z, Pourseyedi S, Nadernejad N. Investigation of ZnO nanoparticles on proline, anthocyanin contents and photosynthetic pigments and lipid peroxidation in the soybean. IET Nanobiotechnol. 2019;13(1): 66–70. doi: 10.1049/iet-nbt.2018.5212 30964040 PMC8676270

[54] Milewska-Hendel A, Gawecki R, Zubko M, Stróż D, Kurczyńska E. Diverse influence of nanoparticles on plant growth with a particular emphasis on crop plants. Acta Agrobot. 2016;69(4): 1694. doi: 10.5586/aa.1694

[55] Pérez-de-Luque A. Interaction of nanomaterials with plants: What do we need for real applications in agriculture? Front Environ Sci. 2017;5: 12. doi: 10.3389/fenvs.2017.00012

[56] Hossain Z, Yasmeen F, Komatsu S. Nanoparticles: synthesis, morphophysiological effects, and proteomic responses of crop plants. Int J Mol Sci. 2020;21(9): 3056. doi: 10.3390/ijms21093056 32357514 PMC7246787

[57] Xiong D, Chen J, Yu T, Gao W, Ling X, Li Y, et al. SPAD-based leaf nitrogen estimation is impacted by environmental factors and crop leaf characteristics. Sci Rep. 2015;5: 13389. doi: 10.1038/srep13389 26303807 PMC4548214

[58] Kaur S, Tiwari V, Kumari A, Chaudhary E, Sharma A, Ali U, et al. Protective and defensive role of anthocyanins under plant abiotic and biotic stresses: An emerging application in sustainable agriculture. J Biotech. 2023;361: 12–29. doi: 10.1016/j.jbiotec.2022.11.009 36414125

[59] Tighe-Neira R, Carmora E, Recio G, Nunes-Nesi A, Reyes-Diaz M, Alberdi M, et al. Metallic nanoparticles influence the structure and function of the photosynthetic apparatus in plants. Plant Physiol Biochem. 2018;130: 408–417. doi: 10.1016/j.plaphy.2018.07.024 30064097

[60] Maxwell K, Johnson GN. Chlorophyll fluorescence–a practical guide. J Exp Bot. 2000; 51(345): 659–668. doi: 10.1093/jxb/51.345.659 10938857

[61] Mujib A, Fatima S, Malik MQ. Cryo-derived plants through embryogenesis showed same levels of vinblastine and vincristine (anticancer) in *Catharanthus roseus* and had normal genome size. Sci Rep. 2022;12: 16635. doi: 10.1038/s41598-022-20993-z 36198853 PMC9534890

[62] Mikuła A, Olas M, Śliwińska E, Rybczyński JJ. Cryopreservation by encapsulation of Gentiana spp. cell suspension maintains regrowth, embryogenic competence and DNA content. CryoLett. 2008; 29(5): 409–418. 18946555

[63] Shukla RK, Badiye A, Vajpayee K, Kapoor N. Genotoxic potential of nanoparticles: structural and functional modifications in DNA. Front Genet. 2021;12: 728250. doi: 10.3389/fgene.2021.728250 34659351 PMC8511513

[64] Abdelsalam NR, Abdel-Megeed A, Ali HM, Salem MZM, Al-Hayali MFA, Elshikh MS. Genotoxicity effects of silver nanoparticles on wheat (*Triticum aestivum* L.) root tip cells. Ecotoxicol Environ Saf 2018;155: 76–85. doi: 10.1016/j.ecoenv.2018.02.069 29510312

[65] Patlolla AK, Berry A, May L, Tchounwou PB. Genotoxicity of silver nanoparticles in *Vicia faba*: a pilot study on the environmental monitoring of nanoparticles. Int J Environ Res Public Health. 2012;9: 1649. doi: 10.3390/ijerph9051649 22754463 PMC3386578

[66] Li K, Zhao X, Hammer BK, Du S, Chen Y. Nanoparticles inhibit DNA replication by binding to DNA: Modeling and experimental validation. ACS Nano. 2013;7(11): 9664–9674. doi: 10.1021/nn402472k 24093667

[67] Kulus D, Tymoszuk A, Jędrzejczyk I, Winiecki J. Gold nanoparticles and electromagnetic irradiation in tissue culture systems of bleeding heart: biochemical, physiological, and (cyto)genetic effects. PCTOC. 2022;149: 715–734. doi: 10.1007/s11240-022-02236-1

[68] Kokina I, Jahundoviča I, Mickeviča I, Sledevskis E, Ogurcovs A, Polyakov B, et al. The impact of CdS nanoparticles on ploidy and DNA damage of rucola (*Eruca sativa* Mill.) plants. J Nanomater. 2015;vol. 2015: Article ID 470250, 7 pages. doi: 10.1155/2015/470250

[69] Prokhorova IM, Kibrik BS, Pavlov AV, Pesnya DS. Estimation of mutagenic effect and modifications of mitosis by silver nanoparticles. Bull Exp Biol Med. 2013;156(2): 255–259. doi: 10.1007/s10517-013-2325-8 24319763

[70] Mehrian SK, Lima RD. Nanoparticles cyto and genotoxicity in plants: Mechanisms and abnormalities. Environ Nanotechnol Monit Manag. 2016;6: 184–193. doi: 10.1016/j.enmm.2016.08.003

[71] Castillo-González J, Ojeda-Barrios D, Hernández-Rodríguez A, González-Franco AC, Robles-Hernández L, López-Ochoa GR. Zinc metalloenzymes in plants. Interciencia. 2018;43(4): 242–248.

[72] Ishido M, Suzuki T, Adachi T, Kunimoto M. Zinc stimulates DNA synthesis during its antiapoptotic action independently with increments of an antiapoptotic protein, Bcl-2, in porcine kidney LLC-PK(1) cells. J Pharmacol Exp Ther. 1999;290(2): 923–8. 10411610

[73] Hao YJ, You CX, Deng XX. Analysis of ploidy and the patterns of amplified fragment length polymorphism and methylation sensitive amplified polymorphism in strawberry plants recovered from cryopreservation. CryoLett. 2002;23(1): 37–46. 11912506

[74] Fernandes P, Rodriguez E, Pinto G, Roldán-Ruiz I, De Loose M, Santos C. Cryopreservation of *Quercus suber* somatic embryos by encapsulation-dehydration and evaluation of genetic stability. Tree Physiol. 2008;28(12): 1841–50. doi: 10.1093/treephys/28.12.1841 19193567

