# Supplementary figures and images for: Effect of nanoparticles on the ex-vitro performance of cryopreservation-derived plant material
Source: PLoS One. 2024 Sep 12;19(9):e0310424. doi: 10.1371/journal.pone.0310424 (PMC11392386; doi:10.1371/journal.pone.0310424)

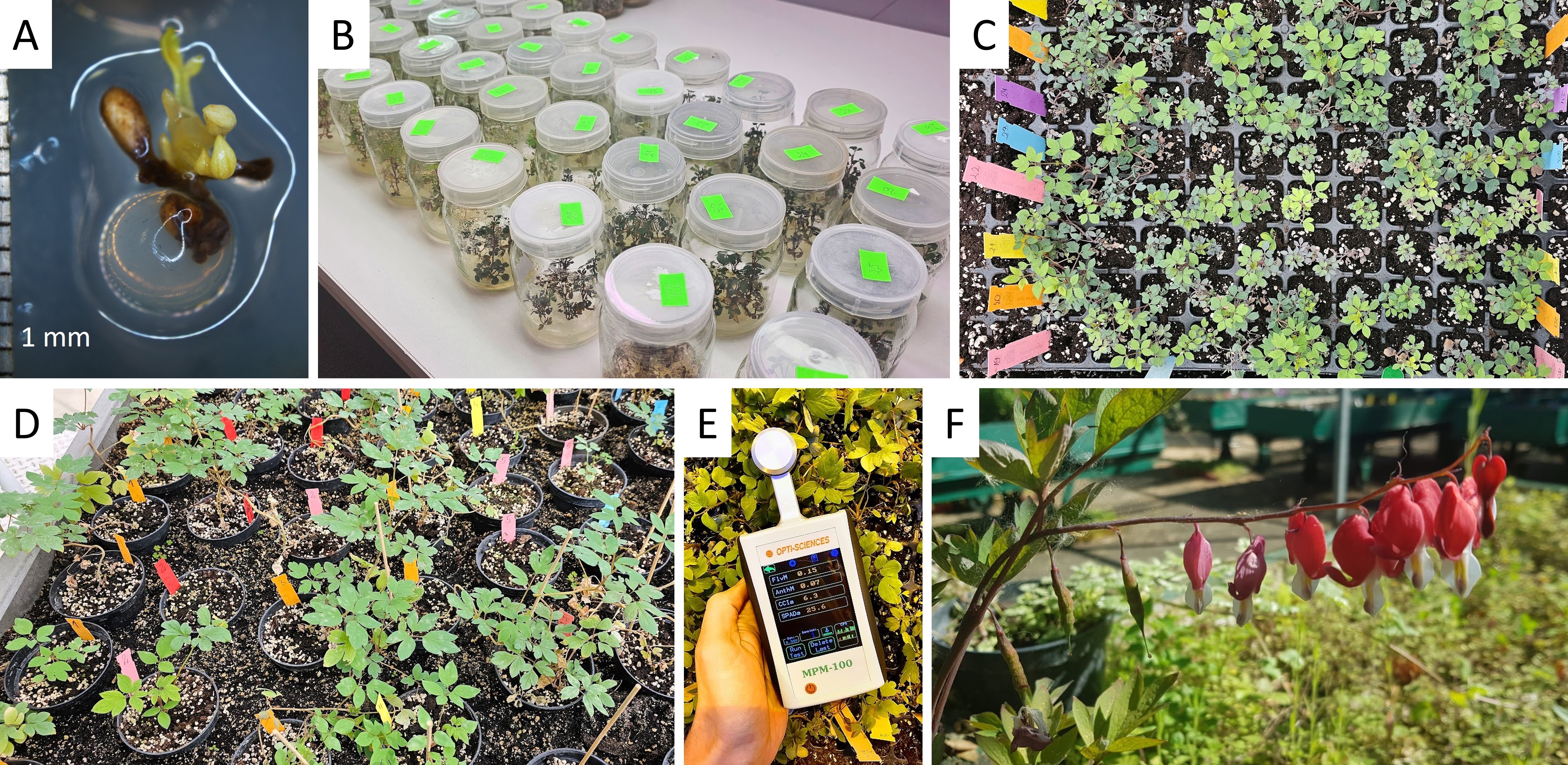

Supplement: S1 Fig — A–developing shoot breaking the alginate capsule; B–in vitro-recovered plantlets; C–acclimatization in a multipot; D–vegetative growth of plants in a glasshouse; E–measurement of pigment content in leaves; F–flowering plant with no signs of phenotype variation. (JPG) [file pone.0310424.s001.jpg]
